# GATA1 Transcriptionally Upregulates LMCD1, Promoting Ferroptosis in Sepsis‐Associated Acute Kidney Injury Through the Hippo/YAP Pathway

**DOI:** 10.1002/kjm2.70071

**Published:** 2025-07-12

**Authors:** You‐Qi Zhang, Wei‐Xia Peng, You‐Fu Li, Peng Lu, Hui Liu, Wen‐Guang Liu

**Affiliations:** ^1^ Department of Intensive Care Unit Yiyang Central Hospital Yiyang Hunan Province People's Republic of China; ^2^ Department of Endocrinology Yiyang Central Hospital Yiyang Hunan Province People's Republic of China; ^3^ Department of Emergency Yiyang central Hospital Yiyang Hunan Province People's Republic of China; ^4^ Department of Respiratory Medicine Yiyang central Hospital Yiyang Hunan Province People's Republic of China

**Keywords:** CUL3, ferroptosis, GATA1, LMCD1, sepsis‐associated acute kidney injury

## Abstract

Sepsis‐associated acute kidney injury (SA‐AKI) is associated with morbidity and mortality. Ferroptosis is associated with the pathophysiology of SA‐AKI. Here, we investigated the role of LIM and cysteine‐rich domain 1 (LMCD1) in the regulation of ferroptosis during SA‐AKI progression. SA‐AKI models were established by CLP and LPS treatments. Hematoxylin and eosin (HE) and (PAS) staining were conducted to examine renal pathological changes. ELISA was used to measure serum creatinine and BUN levels. Lipid ROS levels in cells were examined using flow cytometry. MDA, GSH, SOD, and Fe^2+^ levels in the cells were measured using appropriate kits. Molecular interactions were analyzed using ChIP, a dual‐luciferase reporter gene, and Co‐IP assays. GATA1 knockdown inhibits LPS‐induced cell injury, oxidative stress, and ferroptosis in HK‐2 cells by transcriptionally inhibiting LMCD1 expression. Moreover, LMCD1 activates the Hippo/YAP pathway by promoting CUL3‐mediated Nrf2 ubiquitination degradation. LMCD1 knockdown alleviates CLP‐induced AKI in mice by inhibiting ferroptosis. Taken together, LMCD1 transcriptionally activated by GATA1 promotes ferroptosis during SA‐AKI progression by activating the Hippo/YAP pathway and facilitating CUL3‐mediated Nrf2 ubiquitination degradation.

**Abbreviations**
CCK‐8: cell counting kit‐8
ChIP: chromatin immunoprecipitation
CLP: cecal ligation and puncture
Co‐IP: coimmunoprecipitation
CUL3: Cullin 3
ELISA: enzyme linked immunosorbent assay
GATA1: GATA binding protein 1
GSH: glutathione
HE: hematoxylin‐eosin
LMCD1: LIM and cysteine‐rich domain 1
LPS: lipopolysaccharide
MDA: malondialdehyde
Nrf2: nuclear factor erythroid 2‐related factor 2
PAS: periodic acid‐Schiff
qRT‐PCR: quantitative real‐time polymerase chain reaction
ROS: reactive oxygen species
RTEC: renal tubular epithelial cell
SA‐AKI: Spsis‐associated acute kidney injury
SOD: superoxide dismutase
YAP: yes‐associated protein

## 1 Introduction

Sepsis is a condition of organ failure caused by a dysregulated infection response that can be fatal in extreme situations [1]. Sepsis and acute kidney injury have long been recognized as serious diseases, with fatality rates [2]. As reported, approximately 40% of sepsis patients have AKI [3], and the death rate among sepsis‐associated acute kidney injury (SA‐AKI) patients can reach 70% [4]. The pathophysiology of SA‐AKI is complex, and no specific medications or procedures are currently available. Therefore, there is an urgent need to develop innovative treatment techniques for SA‐AKI, and understanding the pathogenesis of SA‐AKI is the key to achieving this goal. Ferroptosis is a new type of programmed cell death that requires iron. It is a key mechanism of continuous renal tubular epithelial cell (RTEC) death in SA‐AKI, and its inhibition can reduce the severity of kidney injury [5]. Therefore, inhibition of ferroptosis in RTECs may be a potential therapeutic strategy for SA‐AKI.

LIM and cysteine‐rich domain 1 (LMCD1) are transcriptional cofactors containing four main structural domains that play key roles in various diseases, including kidney injury [6]. LMCD1 is upregulated in the kidney tissues of patients with chronic kidney disease, and its silencing ameliorates renal fibrosis in mice [7]. It has also been revealed that LMCD1 is a potential genetic contributor to AKI [8]. However, the role of LMCD1 in SA‐AKI remained unclear. In the present study, we predicted that LMCD1 interacts with Cullin 3 (CUL3) using the BioGRID database. CUL3 is a ubiquitin ligase [9]. The disruption of CUL3‐mediated ubiquitination exacerbates AKI by promoting kidney fibrosis [10]. Notably, CUL3 was highly expressed in SA‐AKI and lipopolysaccharide (LPS)‐treated mouse podocytes [11]. In addition, CUL3 triggers oxidative damage and cell injury by promoting polyubiquitination of nuclear factor erythroid 2‐related factor 2 (Nrf2) and inhibiting Nrf2 activity [12]. Nrf2 can promote cell survival after oxidative damage by regulating downstream signaling pathways, such as the Hippo/Yes‐associated protein (YAP) pathway [13]. It has been reported that Nrf2 inhibits ferroptosis under pathological conditions by inactivating the Hippo/YAP signaling [14]. Therefore, we concluded that CUL3 may promote ferroptosis during SA‐AKI progression by activating Hippo/YAP signaling and promoting Nrf2 ubiquitination and degradation.

Finally, we investigated the mechanisms upstream of LMCD1 in the regulation of SA‐AKI progression. Transcription factors play important roles in disease by transcriptionally regulating downstream genes transcriptionally [15]. GATA binding protein 1 (GATA1) is a key hematopoietic transcription factor that is crucial for the specification of red blood cells in early hematopoietic stem cells and progenitor cells, containing two zinc finger domains [16]. GATA1 promotes the secretion of pro‐inflammatory cytokines by mediating the transcription of nitric oxide synthase 2 [17]. Inflammation is a common pathological and physiological manifestation of clinical SA‐AKI. Notably, it has been previously described that GATA1 upregulation enhanced hypoxia/reperfusion‐induced inflammatory responses in HK‐2 cells [18]. GATA1 plays a role in illness via the transcriptional regulation of downstream targets. Evidence suggests that GATA1 promotes the development of ovarian cancer by transcriptionally increasing JAG1 expression [19]. Additionally, GATA1 repressed human coronary artery endothelial cell pyroptosis by activating RORalpha transcription [20]. Using the JASPAR database, the present study suggested that GATA1 has possible binding sites for the LMCD1 promoter. Nevertheless, the interaction between GATA1 and LMCD1 in the regulation of ferroptosis during development is unclear and warrants further investigation.

Collectively, it is speculated that LMCD1 transcriptionally activated by GATA1 promotes ferroptosis in RTECs during SA‐AKI progression by activating Hippo/YAP signaling and promoting Nrf2 ubiquitination degradation. Our study suggests that LMCD1 is a potential diagnostic and therapeutic target for SA‐AKI.

## 2 Materials and Methods

### 2.1 Cell Culture and Treatment

HK‐2 cells were obtained from ATCC (VA, USA) and grown in DMEM/F‐12 (Gibco, MD, USA) containing 10% FBS (Gibco) with 5% CO_2_ at 37°C. HK‐2 cells were incubated with different concentrations of LPS (0, 5, 10, 20, and 40 μg/mL) for 22 h. HK‐2 cells were incubated with 2 μM Fer‐1 (ferroptosis inhibitor; Sigma‐Aldrich) for 16 h.

### 2.2 Cell Transfection

GenePharma (Shanghai, China) provided short hairpin RNAs (sh‐GATA1, sh‐LMCD1, and sh‐CUL3), overexpression plasmids (Oe‐LMCD1, Oe‐GATA1, and Oe‐CUL3) and negative controls. The LMCD1 shRNA fragment was cloned into the AAV vector GV478 (GeneChem, Shanghai, China) to generate AAV‐LMCD1. Cells were transfected with plasmids and shRNAs using Lipofectamine 3000 (Invitrogen).

### 2.3 Cell Counting Kit‐8 (CCK‐8) Assay

Cells were seeded into 24‐well plates (2 × 10^4^ cells/well) and cultured for 24 h. Cells were incubated for 3 h with CCK‐8 solution (10 μL; Solarbio, Beijing, China) at 37°C. The absorbance was measured at 450 nm.

### 2.4 Lipid Reactive Oxygen Species (ROS) Detection

After different treatments, HK‐2 cells were incubated with 10 μM C11 BODIPY 581/591 (Invitrogen, CA, USA) for 1 h. Cells were subsequently collected and analyzed by flow cytometry (BD. NJ, USA).

### 2.5 Chromatin Immunoprecipitation (ChIP) Assay

Cells were fixed for 10 min in 1% formaldehyde solution, and the fragmented chromosomes (200–1000 bp) were first created through ultrasound. The samples were treated overnight with anti‐GATA1 (Abcam, Cambridge, UK, 1:100, ab181544) or anti‐IgG (Abcam, 1:100, ab172730) at 4°C. DNA was immunoprecipitated using protein A/G beads (Sigma‐Aldrich), and the precipitated DNA was analyzed using qPCR.

### 2.6 Dual Luciferase Reporter Gene Assay

Potential binding sites between the GATA1 and LMCD1 promoters were predicted using the JASPAR database (https://jaspar.elixir.no/). The LMCD1 promoter fragment was amplified using PCR, and the GATA1 binding site in the fragment was mutated. WT and MUT LMCD1 sequences were cloned into the psicheck2 vector (Promega, Madison, WI, USA). The cells were then co‐transfected with the WT or MUT plasmid and Oe‐GATA1 or vector. Luciferase activity was measured 48 h after induction.

### 2.7 Coimmunoprecipitation (Co‐IP)

HK‐2 cells were lysed in a lysis buffer containing protease inhibitors. The cell lysates were incubated with the primary antibody against IgG (Abcam, 1:100, ab172730), LMCD1 (Abcam, 1:50, ab179454), or CUL3 (Abcam, 1:50, ab75851) at 4°C overnight and incubated with sepharose CL‐4B beads (Sigma‐Aldrich) at 4°C for 4 h. Bound proteins were eluted from the protein–antibody complexes and used for western blot analysis.

### 2.8 Ubiquitination Assay

The cell lysates were sonicated and incubated with anti‐IgG (Abcam, 1:100, ab172730) or anti‐Nrf2 (Abcam, 1:200, ab137550) for 3 h at 4°C. The samples were then treated for 4 h with protein A/G IP magnetic beads (Sigma‐Aldrich) at 4°C. Nrf2 ubiquitination levels were determined by western blot using an anti‐Ub antibody (Abcam, 1:1000, ab140601).

### 2.9 Animal Experiments

SJA (Hunan, China) provided 42 C57BL/6J mice (6–8‐week‐old, 20–22 g), which were randomly divided into sham, CLP, CLP + AAV‐NC, CLP + AAV‐LMCD1, CLP + Resatorvid, CLP + AFM32a, and CLP + DEX groups (*n* = 6). SA‐AKI mice were established by cecal ligation and puncture (CLP) according to a previous study [21]. Briefly, the mice were sedated with a sodium pentobarbital (60 mg/mL) injection before a midline abdominal incision was made. Ligation of the ileocecal valve was performed at the distal 3/4 of the cecum. Polymicrobial peritonitis was induced by perforating the cecum twice with a 21‐gauge needle and squeezing out a drop of feces from the perforation site. Two layers were sutured along the abdominal wall, and 1 mL of 0.9% sodium chloride solution was administered for liquid resuscitation. The rats in the sham group underwent laparotomy and intestinal manipulation without ligation or perforation. To knock down LMCD1 in vivo, mice were injected with AAV‐LMCD1 or AAV‐NC (2 × 10^10^ v.g./mL) via the visual tail vein method 7 days before CLP. For the intervention groups, resatorvid (a TLR4 inhibitor, 1 mg/kg; Selleck Chemicals, TX, USA) was administered intraperitoneally once daily for 7 days, starting 1 h prior to CLP. PAD2 inhibitor (AFM32a, 20 mg/kg, MCE, NJ, USA) dissolved in DMSO (1 μL per kg mouse body weight) was given intraperitoneally 1 h after CLP. Dexamethasone (DEX, 1 mg/kg; Sigma‐Aldrich) was administered via intraperitoneal injection once daily for 7 days, beginning 24 h prior to CLP. The mice were euthanized after 24 h, and kidney tissues and blood were collected. All animal studies were approved by the Yiyang Central Hospital.

### 2.10 Enzyme Linked Immunosorbent Assay (ELISA)

Serum levels of serum creatinine (Scr), blood urea nitrogen (BUN), and LPS were determined using kits purchased from StressMarq Biosciences (BC, Canada; #SKT‐217, #SKT‐213, #SKT‐210). The results were recorded by measuring the OD value at 450 nm.

### 2.11 Hematoxylin–Eosin (HE) Staining

The kidney tissues were fixed for 72 h in 4% paraformaldehyde, gradient dealcoholized with xylene, and eluted with water. The 5 μm‐thick paraffin sections were prepared, eluted in a reverse gradient using ethanol and water, and stained with HE (Sigma‐Aldrich). The sections were observed and photographed using an Olympus microscope (Tokyo, Japan). Histological changes were assessed in a double‐blind manner in five different regions randomly selected from each sample.

### 2.12 Periodic Acid‐Schiff (PAS) Staining

The kidney tissue paraffin sections (5 μm) were stained with PAS solution (Sigma‐Aldrich). All images were captured using an Olympus microscope. The degree of glomerular injury by PAS staining was assessed using a scoring system, with scores ranging from 0 to 4 points, based on injury level (< 25%, 25%–50%, 50%–75%, and > 75%).

### 2.13 Measurement of ROS Level in Kidney Tissues

ROS levels in the kidney tissues were examined using a ROS assay kit (E004‐1‐1, Jiancheng, Jiangsu, China) according to the manufacturer's instructions.

### 2.14 Measurement of Fe^2+^, Malondialdehyde (MDA), Glutathione (GSH), and Superoxide Dismutase (SOD) Levels

Fe^2+^, MDA, MPO, and GSH levels were examined using Fe^2+^ (MAK025, Sigma‐Aldrich), MDA (A003‐1‐2, Jiancheng), GSH (A006‐2‐1, Jiancheng), and SOD assay kits (A001‐3‐2, Jiancheng), respectively, according to the manufacturer's instructions.

### 2.15 Quantitative Real‐Time Polymerase Chain Reaction (qRT‐PCR)

Total RNA was extracted from tissues and cells using TRIzol reagent (Invitrogen). cDNA was synthesized using a cDNA reverse transcription kit (TaKaRa, Tokyo, Japan). qRT‐PCR was performed using SYBR reagent (TaKaRa). RNA expression was calibrated using GAPDH as the reference gene. The primers used were listed as follows (5′‐3′):

LMCD1 human (F): GGTGGCTAAGGACCTCAACC,

LMCD1 human (R): GTCCCCTTGCATCCCAAAC;

LMCD1 mouse (F): ATGGCAAAAGTGGCTAAGGAC,

LMCD1 mouse (R): CCACGAGTGAGGCTCGAAG;

GATA1 human (F): CTGTCCCCAATAGTGCTTATGG,

GATA1 human (R): GAATAGGCTGCTGAATTGAGGG;

GAPDH human (F): AGGTCGGTGTGAACGGATTTG,

GAPDH human (R): GGGGTCGTTGATGGCAACA;

GAPDH mouse (F): AGGTCGGTGTGAACGGATTTG,

GAPDH mouse (R): GGGGTCGTTGATGGCAACA.

### 2.16 Western Blot Analysis

Total protein was extracted from the cells using RIPA (Beyotime). Subsequently, the total protein was separated using 10% SDS‐PAGE polyacrylamide gels. Following electrophoresis and membrane transfer, the PVDF membrane (MA, USA) was used. The membranes were then blocked and incubated overnight with antibodies against LMCD1 (Thermofisher, 1:1000, PA5‐49414), GATA1 (Abcam, 1:1000, ab40847), YAP (Abcam, 1:1000, ab205270), p‐YAP (Abcam, 1:10000, ab76252), GPX4 (Abcam, 1:5000, ab125066), SLC7A11 (Abcam, 1:1000, ab307601), ACSL4 (Abcam, 1:10000, ab155282), Nrf2 (Abcam, 1:1000, ab62352), and β‐actin (Abcam, 1:5000, ab8226). The membranes were then incubated for 60 min with the secondary antibody (ab7090, 1:5000, Abcam). Protein bands were visualized using enhanced chemiluminescence (Beyotime) and quantified using ImageJ.

### 2.17 Data Analysis

All experiments were independently conducted in triplicate. All the data were statistically analyzed with SPSS 19.0 and presented as means ± SD. Unpaired Student's *t*‐test (for two groups) or one‐way ANOVA followed by Tukey's post hoc test (for more than two groups) was performed to calculate the statistical values. Statistical *p* values were less than 0.05, significant.

## 3 Results

### 3.1 LMCD1 Knockdown Alleviated LPS‐Induced Oxidative Stress and Ferroptosis in RTECs

**FIGURE 1:**
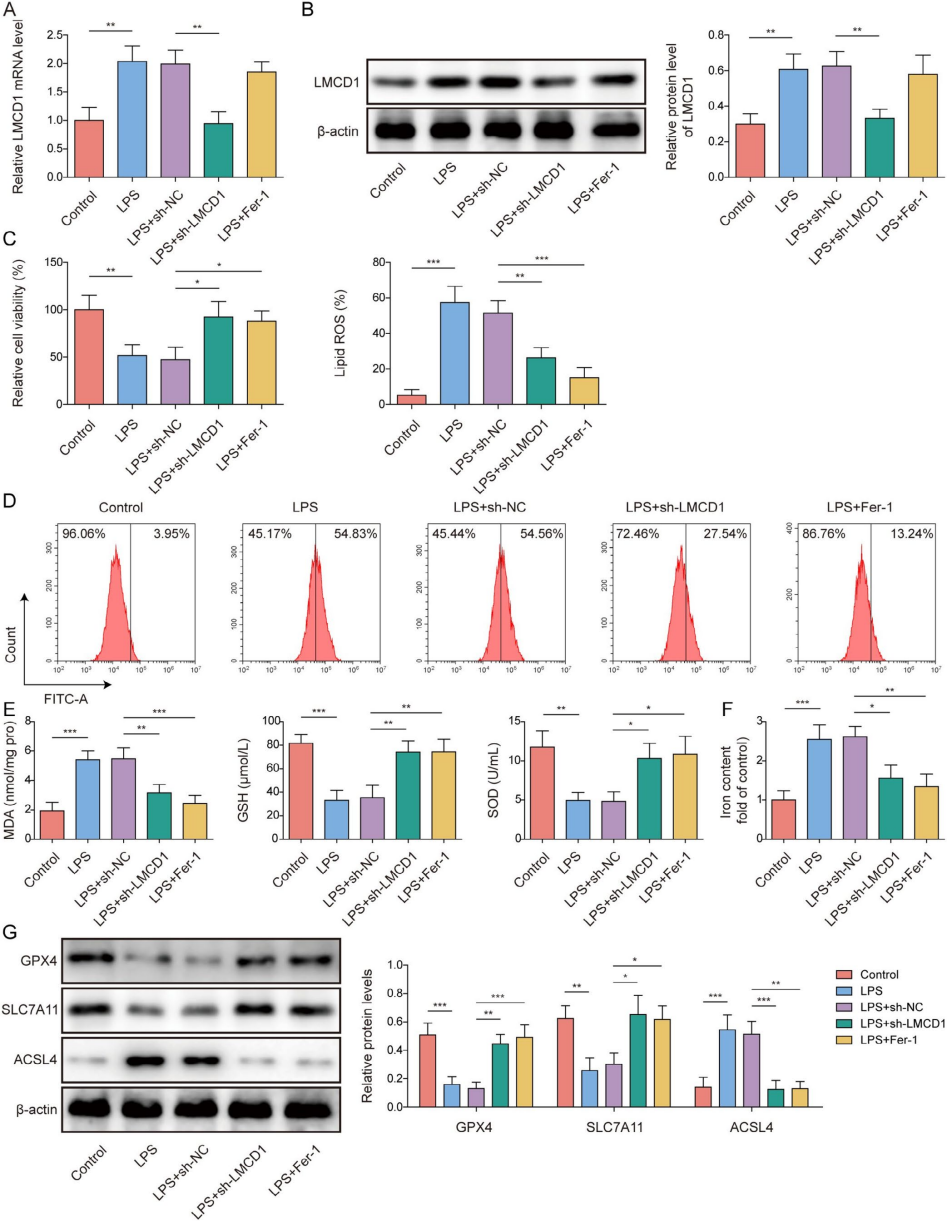
LMCD1 knockdown alleviated LPS‐induced oxidative stress and ferroptosis in RTECs. The SA‐AKI cell model was established by LPS treatment and then transfected with sh‐LMCD1 or sh‐NC or a ferroptosis inhibitor (Fer‐1). (A, B) LMCD1 expression levels were determined by qRT‐PCR and western blotting. (C) HK‐2 cell viability was examined using the CCK‐8 assay. (D) Lipid ROS levels in cells were examined by flow cytometry. (E, F) MDA, GSH, SOD, and Fe^2+^ levels in the cells were analyzed using appropriate kits. (G) Western blotting was performed to measure SLC7A11, GPX4, and ACSL4 levels in the cells. The measurement data were presented as mean ± SD. All the data were obtained from at least three replicates. *p < 0.05, **p < 0.01, ***p < 0.001.

As previously reported, LMCD1 upregulation promotes renal fibrosis in mice [7]. However, the role of LMCD1 in SA‐AKI remained unclear. Oxidative stress and ferroptosis are overactivated during SA‐AKI development, and the inhibition of ferroptosis can alleviate kidney injury [5]. To study the significance of LMCD1 in regulating oxidative stress and ferroptosis during SA‐AKI progression, a cell model of SA‐AKI was established by LPS treatment and then transfected with sh‐LMCD1/sh‐NC or ferroptosis inhibitor (Fer‐1). We tested different LPS concentrations (0, 5, 10, 20, and 40 μg/mL) on HK‐2 cells. It was found that 10 μg/mL LPS significantly reduced HK‐2 cell viability and decreased LMCD1 mRNA level in cells (Figure S1A,B). Previous studies have also used 10 μg/mL LPS to establish the SA‐AKI cell model [22, 23]. Therefore, this concentration was selected for the subsequent experiments. As shown in Figure 1A,B, LPS treatment significantly elevated LMCD1 mRNA and protein levels in HK‐2 cells; however, this effect was weakened by sh‐LMCD1 transfection, and Fer‐1 treatment did not affect LMCD1 expression. HK‐2 cell viability was impaired by LPS stimulation, whereas LMCD1 knockdown and Fer‐1 treatment partially reversed this effect (Figure 1C). In addition, LPS treatment significantly increased lipid ROS, MDA, and Fe^2+^ levels, while reducing GSH and SOD levels in HK‐2 cells, which was abolished by LMCD1 silencing or Fer‐1 treatment (Figure 1D–F). Finally, LPS treatment significantly reduced the protein levels of ferroptosis suppressor proteins (GPX4 and SLC7A11) and elevated the levels of a ferroptosis protein (ACSL4) in HK‐2 cells, whereas these changes were weakened by LMCD1 knockdown or Fer‐1 treatment (Figure 1G). Collectively, LMCD1 silencing markedly attenuates LPS‐induced HK‐2 cell injury, oxidative stress, and ferroptosis.

### 3.2 GATA1 Activated the Hippo/YAP Pathway by Transcriptionally Upregulating LMCD1

**FIGURE 2:**
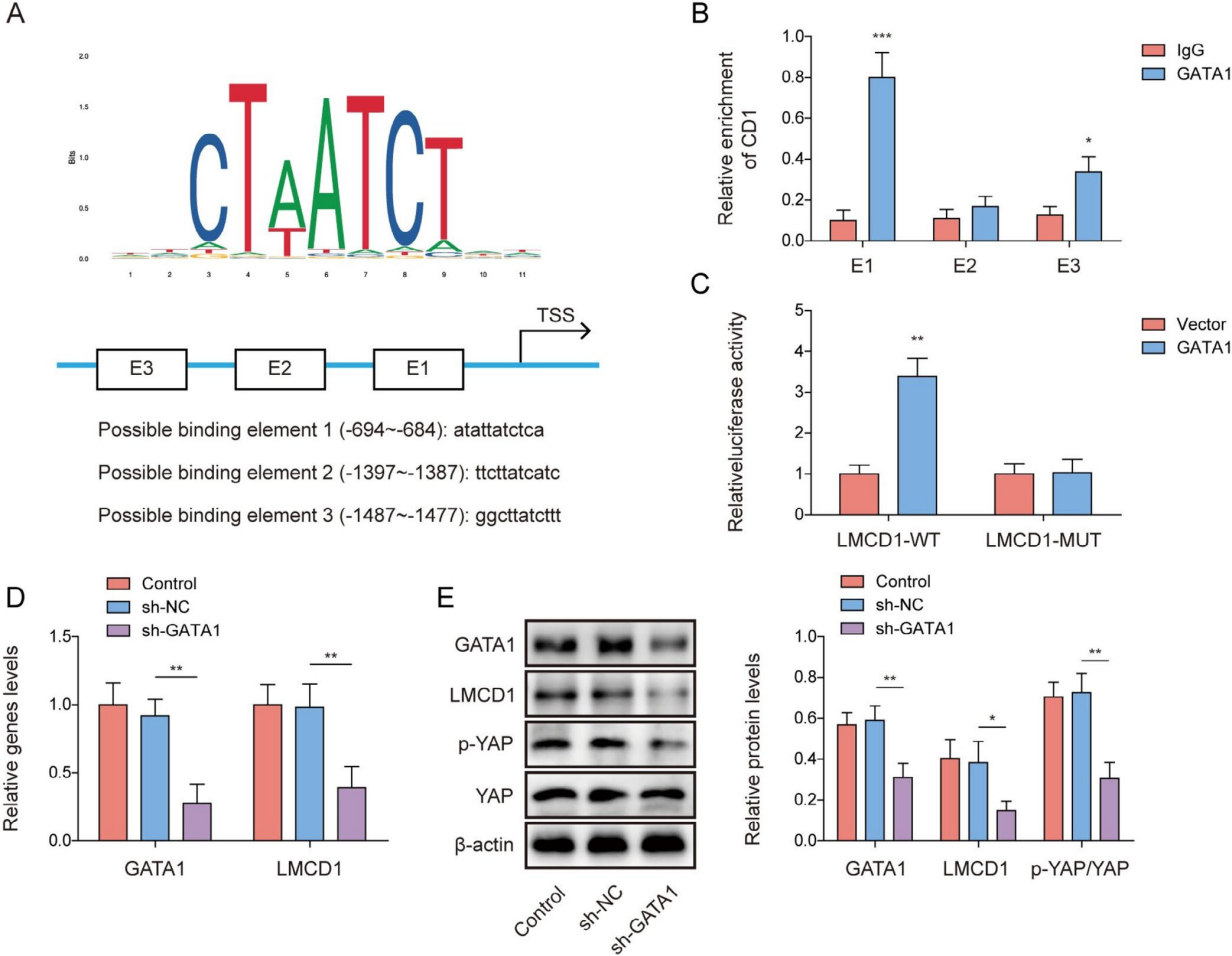
GATA1 activated the Hippo/YAP pathway by transcriptionally upregulating LMCD1. (A) The potential binding sites between GATA1 and LMCD1 promoter was predicted using the JASPAR database. (B, C) ChIP and dual luciferase reporter gene assays were employed to analyze the interaction between GATA1 and LMCD1 promoter. HK‐2 cells were transfected with sh‐NC or sh‐GATA1. (D) GATA1 and LMCD1 mRNA levels in cells were detected by qRT‐PCR. (E) Western blot was performed to examine GATA1, LMCD1, p‐YAP, and YAP protein levels in cells. The measurement data were presented as mean ± SD. All data was obtained from at least three replicate experiments. *p < 0.05, **p < 0.01, ***p < 0.001.

We investigated the upstream mechanisms of LMCD1 in the regulation of SA‐AKI development. GATA1 is a hematopoietic transcription factor that promotes ischemia/reperfusion‐induced AKI [18]. As shown in Figure 2A, it was predicted that GATA1 had potential binding sites for the LMCD1 promoter using the JASPAR database. The ChIP assay showed that the GATA1 antibody markedly enriched the LMCD1 at the E1 site (Figure 2B). Meanwhile, it was observed that GATA1 overexpression significantly increased the luciferase activity of LMCD1‐WT but did not affect that of LMCD1‐MUT significantly (Figure 2C). HK‐2 cells were subsequently transfected with sh‐GATA1 or sh‐NC, and sh‐GATA1 transfection significantly reduced GATA1, LMCD1, and p‐YAP levels in HK‐2 cells (Figure 2D,E). Taken together, GATA1 activates Hippo/YAP signaling in renal epithelial cells by transcriptionally activating LMCD1 expression.

### 3.3 GATA1 Regulated LPS‐Induced Oxidative Stress and Ferroptosis in RTECs by Transcriptionally Upregulating LMCD1

**FIGURE 3:**
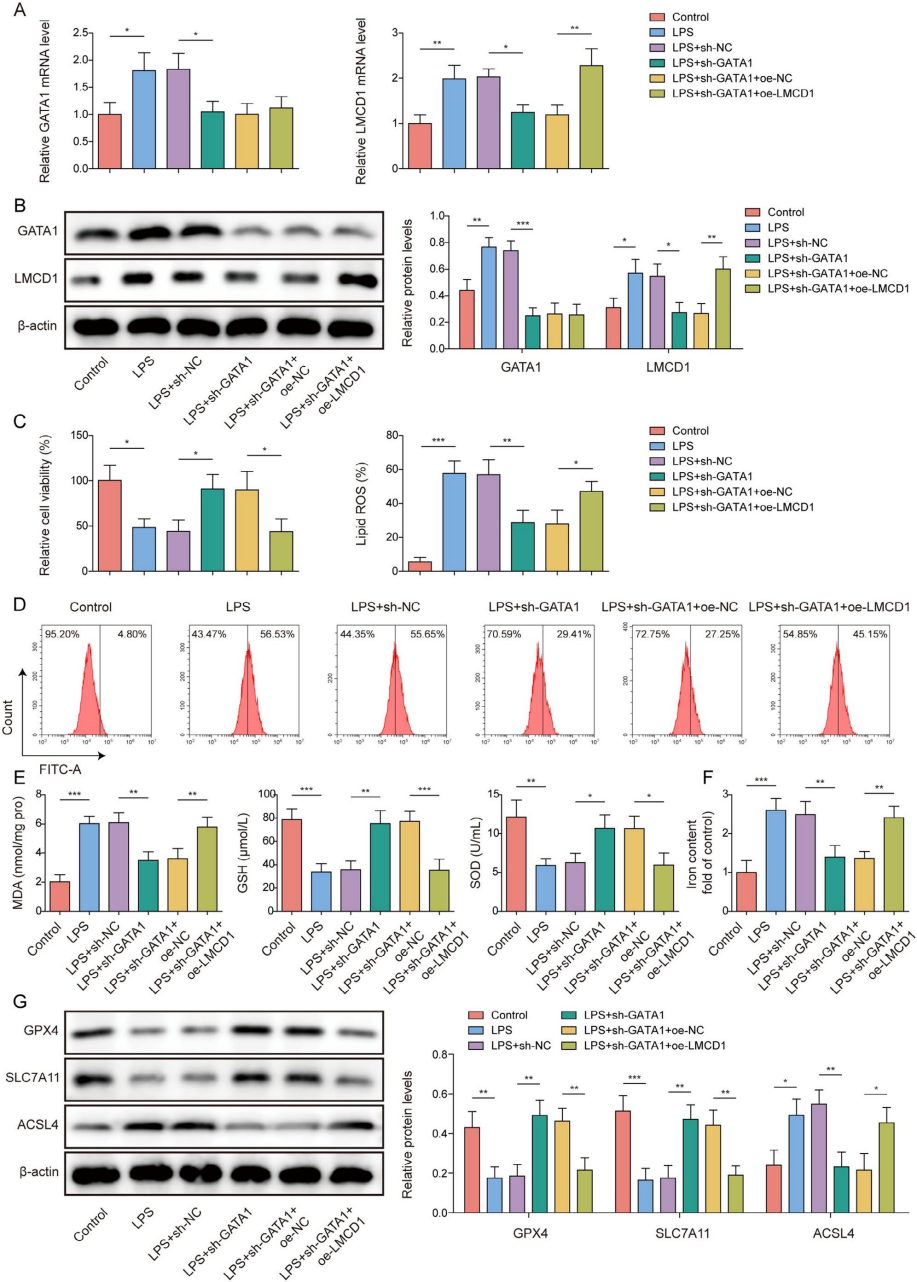
GATA1 regulated LPS‐induced oxidative stress and ferroptosis in RTECs by transcriptionally upregulating LMCD1. Both GATA1 knockdown and LMCD1 overexpression were observed in LPS‐treated HK‐2 cells. (A, B) GATA1 and LMCD1 expression levels were determined using qRT‐PCR and western blotting. (C) CCK‐8 assay was used to detect HK‐2 cell viability. (D) Flow cytometry was performed to detect the lipid ROS levels in the cells. (E‐F) MDA, GSH, SOD, and Fe^2+^ levels in the cells were analyzed using appropriate kits. (G) SLC7A11, GPX4, and ACSL4 protein levels in the cells were analyzed using western blotting. The measurement data were presented as mean ± SD. All the data were obtained from at least three replicates. *p < 0.05, **p < 0.01, ***p < 0.001.

To investigate the roles of GATA1 and LMCD1 in the regulation of oxidative stress and ferroptosis during SA‐AKI progression, GATA1 knockdown and LMCD1 overexpression were induced in LPS‐treated HK‐2 cells. It was initially shown that sh‐GATA1 transfection markedly reduced GATA1 and LMCD1 expression levels in LPS‐induced HK‐2 cells, whereas Oe‐LMCD1 co‐transfection significantly elevated LMCD1 expression but did not significantly affect GATA1 expression levels (Figure 3A,B). In addition, GATA1 silencing markedly increased the viability of LPS‐treated HK‐2 cells, which was attenuated by Oe‐LMCD1 co‐transfection (Figure 3C). Furthermore, GATA1 silencing significantly reduced lipid ROS, MDA, and Fe^2+^ levels and increased GSH and SOD levels in LPS‐treated HK‐2 cells, while these changes were partially eliminated by Oe‐LMCD1 co‐transfection (Figure 3D–F). GATA1 silencing increased GPX4 and SLC7A11 protein levels and reduced ACSL4 levels in LPS‐treated HK‐2 cells; however, these changes were abrogated by LMCD1 overexpression (Figure 3G). Collectively, GATA1 knockdown inhibits LPS‐induced oxidative stress and ferroptosis in RTECs by regulating LMCD1 expression.

### 3.4 LMCD1 Activated the Hippo/YAP Pathway by Promoting Nrf2 Ubiquitination Degradation Through Interacting With CUL3

**FIGURE 4:**
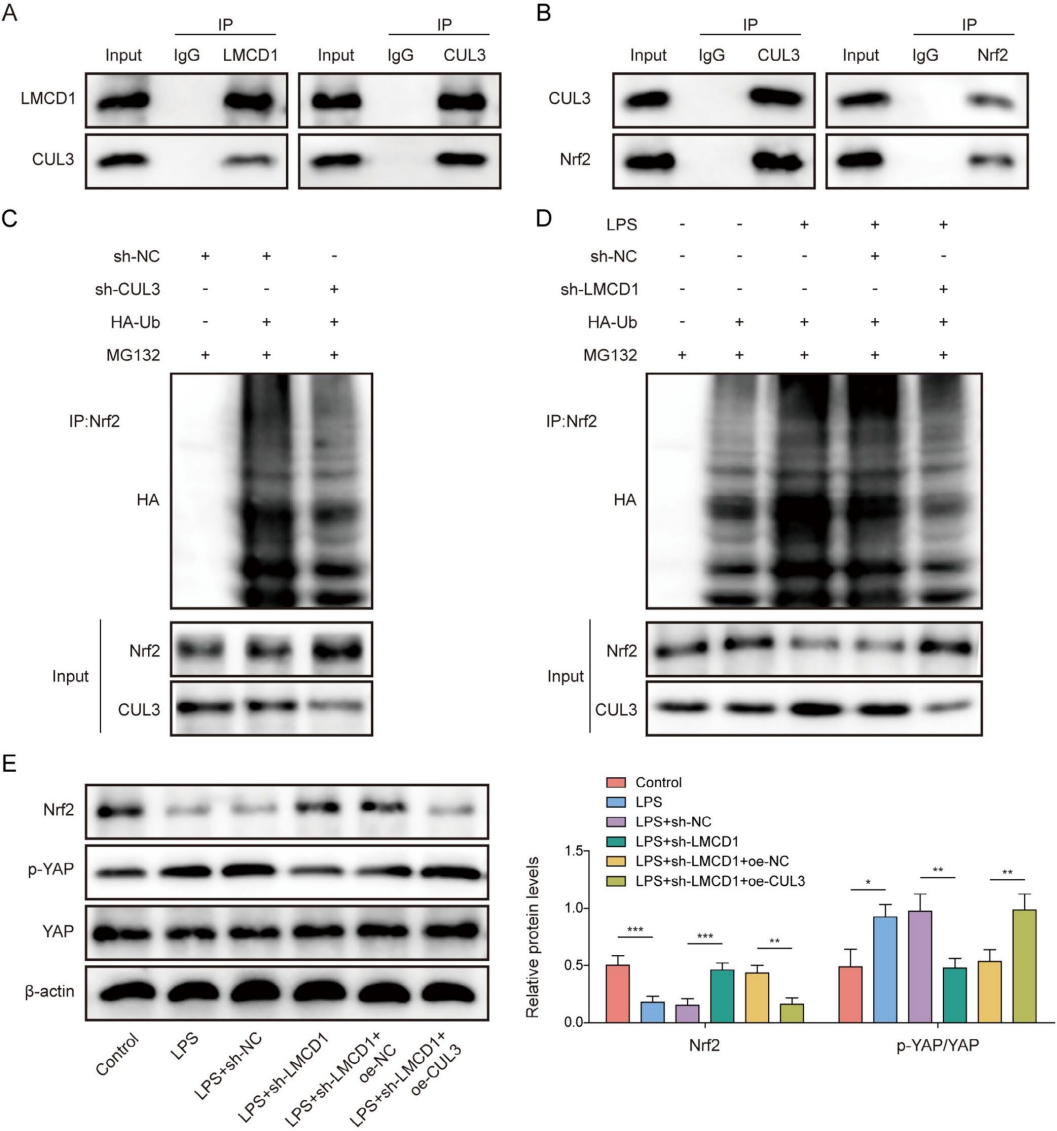
LMCD1 activated the Hippo/YAP pathway by promoting Nrf2 ubiquitination degradation through interacting with CUL3. (A, B) The interactions between LMCD1, CUL3, and Nrf2 were analyzed by Co‐IP assay. (C) HK‐2 cells were transfected with sh‐NC or sh‐CUL3, and Nrf2 ubiquitination level in cells was detected by Co‐IP assay. (D) LPS‐treated HK‐2 cells were transfected with sh‐NC or sh‐LMCD1, and Nrf2 ubiquitination level in cells was detected by Co‐IP assay. (E) Both LMCD1 knockdown and CUL3 overexpression were induced in LPS‐treated HK‐2 cells, and Nrf2, p‐YAP, and YAP protein levels in cells were detected using western blot. The measurement data were presented as mean ± SD. All data was obtained from at least three replicate experiments. *p < 0.05, **p < 0.01, ***p < 0.001.

We investigated the downstream mechanisms of LMCD1 in the regulation of SA‐AKI development. CUL3 is a ubiquitin ligase, and dysregulation of CUL3‐mediated ubiquitination exacerbated AKI [10]. Co‐IP results showed that LMCD1 interacted directly with CUL3 (Figure 4A). As previously described, CUL3 triggers oxidative damage and cell injury by promoting polyubiquitination of Nrf2 [12]. The interaction between CUL3 and Nrf2 was verified using a Co‐IP assay (Figure 4B). Our results demonstrated that CUL3 knockdown reduced Nrf2 ubiquitination levels and increased Nrf2 protein levels in HK‐2 cells (Figure 4C). Additionally, LPS elevated Nrf2 ubiquitination and CUL3 protein levels while reducing Nrf2 protein levels in HK‐2 cells; however, these changes were weakened by LMCD1 silencing (Figure 4D). Finally, LPS reduced Nrf2 protein levels and elevated p‐YAP levels in HK‐2 cells, which were eliminated by LMCD1 silencing. This effect of sh‐LMCD1 was partially reversed after sh‐LMCD1 and Oe‐CUL3 co‐transfection (Figure 4E). Taken together, LMCD1 activates Hippo/YAP signaling by promoting CUL3‐mediated degradation of Nrf2 ubiquitination.

### 3.5 LMCD1 Knockdown Alleviated CLP‐Induced SA‐AKI in Mice

**FIGURE 5:**
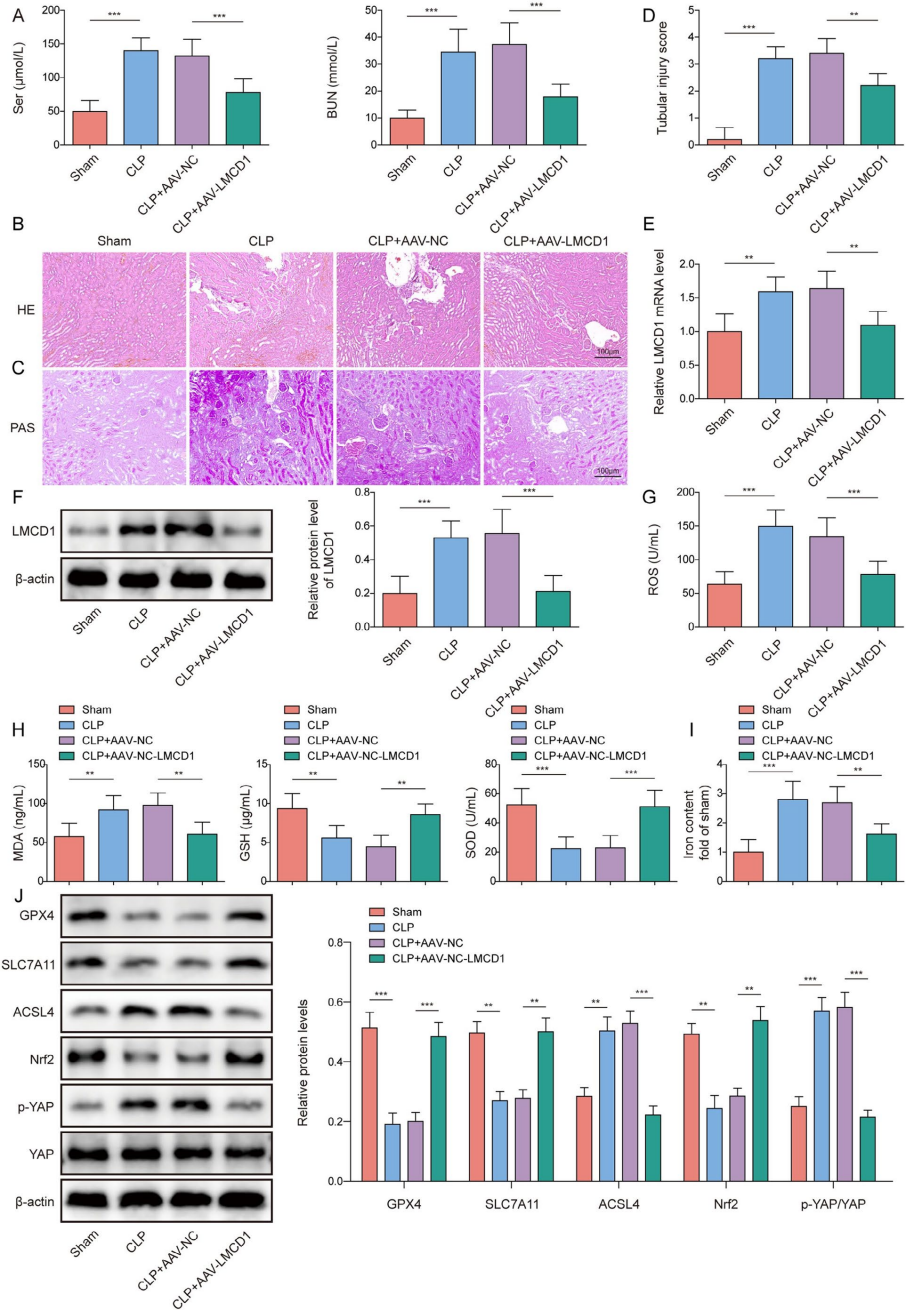
LMCD1 knockdown alleviated CLP‐induced SA‐AKI in mice. SA‐AKI mice were established using CLP and injected with AAV‐LMCD1. (A) Serum creatinine and BUN levels were examined using ELISA. (B‐D) Hematoxylin and eosin (HE) and periodic acid‐Schiff (PAS) staining were performed to analyze the pathological changes in the kidney, and the renal tubular injury score is presented. (E, F) LMCD1 expression levels were determined using qRT‐PCR and western blotting. (G‐I) ROS, MDA, GSH, SOD, and Fe^2+^ levels in the kidney tissues were analyzed using appropriate kits. (J) SLC7A11, GPX4, ACSL4, Nrf2, p‐YAP, and YAP protein levels in kidney tissues were detected using western blotting. *n* = 6. *p < 0.05, **p < 0.01, ***p < 0.001.

SA‐AKI mice were established by CLP. To analyze the biological role of LMCD1 in the regulation of SA‐AKI in vivo, SA‐AKI mice were injected with AAV‐LMCD1. The results demonstrated that CLD increased serum Scr and BUN levels in mice, and these changes were ameliorated by AAV‐LMCD1 injection (Figure 5A). Interestingly, our results showed that CLP treatment significantly increased plasma LPS levels in mice, and this effect was partially reversed by LMCD1 knockdown (Figure S1C). However, the relationship between LPS reduction and ferroptosis remains unclear. It was subsequently observed that CLP treatment resulted in various pathological changes in the kidney, such as tubular epithelial necrosis, tubular structural changes, and increased renal tubular injury scores, and these pathological changes were ameliorated by LMCD1 silencing (Figure 5B–D). In addition, LMCD1 expression levels in kidney tissues were markedly elevated by CLP; however, this effect was partially reversed by AAV‐LMCD1 injection (Figure 5E,F). Moreover, CLP treatment resulted in increased Fe^2+^, MDA, and ROS levels while reducing GSH and SOD levels in the kidneys, but these changes were eliminated by LMCD1 silencing (Figure 5G–I). In this study, we incorporated interventions to reverse LPS‐induced effects in vivo, including TLR4 inhibitors (Resatorvid and TAK‐242), PAD2 inhibitors (AFM32a), and DEX treatment. The findings showed that serum Ser and BUN levels were significantly elevated in the CLP group, while treatment with Resatorvid, AFM32a, or DEX significantly alleviated the increase in these markers, suggesting an improvement in renal function (Figure S1D). Additionally, treatment interventions effectively suppressed ROS and MDA levels while restoring GSH and SOD levels (Figure S1E). Furthermore, the iron content was significantly increased in the CLP group, whereas the intervention treatments effectively reduced the iron content (Figure S1F). We also found that CLP decreased SLC7A11, GPX4, and Nrf2 levels and increased ACSL4 and p‐YAP levels in the kidney tissues, whereas all these changes were weakened by LMCD1 knockdown (Figure 5J). In summary, LMCD1 silencing ameliorates CLP‐induced SA‐AKI in mice by suppressing ferroptosis.

## 4 Discussion

SA‐AKI and its more severe form (end‐stage kidney disease) are devastating diseases that cause substantial morbidity and mortality among patients with sepsis. However, there are no effective or specific treatment strategies for SA‐AKI. The current research supports the hypothesis that inhibiting ferroptosis may help identify novel therapeutic options for SA‐AKI [22]. The main finding of the current study was that LMCD1, transcriptionally activated by GATA1, facilitates ferroptosis in RTECs during SA‐AKI progression by activating Hippo/YAP signaling and enhancing CUL3‐mediated Nrf2 ubiquitination degradation.

LMCD1 contains a LIM domain and is an important regulatory factor in cell fate determination and cell growth. LMCD1 is a novel pre‐transcriptional integrator that links oxidative stress, lipid homeostasis, and cell senescence [24]. Notably, a previous study showed that LMCD1 upregulation promotes unilateral ureteral obstruction‐induced kidney injury by facilitating renal fibrosis and RTEC apoptosis [25]. However, the role of LMCD1 in SA‐AKI remained unclear. Our results showed that LMCD1 expression in HK‐2 cells was significantly elevated by LPS treatment, and its silencing mitigated LPS‐induced oxidative stress and ferroptosis in HK‐2 cells. Additionally, LMCD1 knockdown alleviated CLP‐induced AKI in mice by inhibiting ferroptosis. These results suggest that LMCD1 silencing improves LPS‐induced HK‐2 cell injury and CLP‐induced kidney injury by inhibiting ferroptosis. However, it is important to consider the limitations of the LPS model because it uses a single bacterial strain and cannot accurately replicate the cytokine expression profiles observed in sepsis. Therefore, LPS is no longer considered an effective animal model for simulating sepsis [26]. In contrast, the CLP model better reflects the clinical characteristics of SA‐AKI in humans. LPS is commonly used as an inducer in SA‐AKI cell models, and our findings suggest that LMCD1 is a potential biomarker for sepsis and a target for ferroptosis therapy. This opens up the possibility of LMCD1 serving as a diagnostic and therapeutic target for SA‐AKI. However, the clinical relevance of LMCD1 in human SA‐AKI, particularly its sensitivity and specificity, remains unexplored. Future studies should compare LMCD1 with established clinical biomarkers of SA‐AKI, such as NGAL, IL‐18, and KIM‐1 [27, 28] to evaluate its potential clinical importance. The primary focus of this study was to investigate the mechanistic role of LMCD1 in SA‐AKI, which provides a foundation for further understanding the pathophysiology of the disease and offers promising evidence for the development of diagnostic and therapeutic strategies. Interestingly, we found that LPS levels were elevated under the experimental conditions, while silencing LMCD1 led to a reduction in LPS levels. However, the precise mechanism underlying this phenomenon remains unexplored. Furthermore, whether the observed decrease in LPS levels is linked to LMCD1‐mediated ferroptosis requires further investigation.

The mechanisms upstream of LMCD1 involved in the regulation of SA‐AKI development were also investigated. Transcription factors play a role in the pathophysiology of diseases through transcriptional regulation of target genes. GATA1 is a transcription factor that contains an activation domain [29]. Dysregulated GATA1 expression has been linked to altered lipid metabolism, increased antioxidant activity, and decreased susceptibility to ferroptosis [30]. GATA1 dysregulation also increases the susceptibility to infections and sepsis [31]. However, the role of GATA1 in kidney injury has rarely been studied. Only one study has shown that GATA1 upregulation enhances hypoxia/reperfusion‐induced HK‐2 cell injury by mediating inflammatory responses [18]. Nonetheless, the role of GATA1 in controlling ferroptosis during development is unclear. Our findings illustrated that GATA1 knockdown inhibited LPS‐induced oxidative stress and ferroptosis in HK‐2 cells. GATA1 plays a regulatory role in diseases through transcriptional regulation of target genes. GATA1 induces rheumatoid arthritis development by promoting the transcriptional activation of NOS2 [17]. Our findings reveal that GATA1 activates LMCD1 expression in HK‐2 cells, which has not been previously reported. These results indicated that GATA1 promotes LPS‐induced oxidative stress and ferroptosis in RTECs by transcriptionally elevating LMCD1 expression.

YAP and its paralogs, two Hippo pathway effectors, have recently been shown to be related to sensitivity to ferroptosis [32]. The activation of Hippo/YAP signaling can promote ferroptosis under pathological conditions [33]. Notably, abnormal activation of Hippo/YAP signaling mediates tubular maladaptive repair and promotes inflammation after ischemic AKI [34]. Our results showed that GATA1 activates the Hippo/YAP pathway by transcriptionally upregulating LMCD1 in HK‐2 cells. As previously reported, Nrf2 activation inhibits the phosphorylation and stabilization of YAP [13]. Nrf2 is a key regulatory factor in the cellular antioxidant response, controlling the expression of genes related to resistance to oxidative stress and ferroptosis [35]. As a member of the culin RING family, the CUL3 E3 ligase complex is involved in the ubiquitination of many other proteins [32]. It has been widely reported that CUL3 mediates the ubiquitination and degradation of Nrf2 by interacting with KEAP1 in various diseases [36]. CUL3 expression is closely associated with kidney function. CUL3 disruption in mice unexpectedly causes polyuria and fibrotic injury [37]. Notably, CUL3 dysregulation was critical in SA‐AKI [11]. The function of the CUL3/Nrf2 axis in the regulation of SA‐AKI has not been previously reported. Our current findings illustrate that LMCD1 directly binds to CUL3 and that CUL3 activates Hippo/YAP signaling by promoting the ubiquitination and degradation of Nrf2.

Standard treatment approaches, such as parenteral antibiotic therapy, fluid resuscitation, and administration of vasopressor agents, have not been effective in significantly reducing the incidence of SA‐AKI or its associated mortality [38]. Recombinant proteins and peptides have emerged as promising therapeutic options for various diseases [39]. Based on this, it can be preliminarily suggested that targeting the key proteins involved in this study, such as LMCD1 and GATA1, could provide a potential therapeutic approach for SA‐AKI. By further investigating the roles of these proteins in the pathogenesis of SA‐AKI, it may be possible to develop recombinant proteins that inhibit their expression, potentially offering a new avenue for the treatment of SA‐AKI. Currently, effective and specific interventions to prevent and treat SA‐AKI are lacking. Future studies should focus on understanding the mechanisms underlying SA‐AKI to lay the foundation for the development of diagnostic and therapeutic strategies. Taken together, LMCD1 transcriptionally activated by GATA1 promoted ferroptosis in RTECs during SA‐AKI progression by activating the Hippo/YAP pathway and facilitating CUL3‐mediated Nrf2 ubiquitination and degradation. Our findings provide a theoretical foundation for the development of new treatment options for SA‐AKI.

## Ethics Statement

The animal studies were approved by Yiyang Central Hospital.

## Conflicts of Interest

All authors agree with the presented findings, have contributed to the work, and declare no conflicts of interest.

## Supporting information


**Supporting Information Figure 1.** HK‐2 cells were incubated with different concentrations of LPS (0, 5, 10, 20, and 40 μg/mL) for 22 h. (A) CCK‐8 assay was employed to detect HK‐2 cell viability. (B) LMCD1 mRNA level in cells was detected by qRT‐PCR. All data was obtained from at least three replicate experiments. SA‐AKI mice were established by CLP operation and then were injected with AAV‐LMCD1. (C) Plasma LPS level was measured by ELISA. SA‐AKI mice were established by CLP operation and then subjected to TLR4 inhibitor (Resatorvid, TAK‐242), PAD2 inhibitor (AFM32a), or DEX treatment. (D) Serum Scr and BUN levels were examined using ELISA. (E‐F) ROS, MDA, GSH, SOD, and Fe^2+^ levels in kidney tissues were analyzed by the kits. *n* = 6. The measurement data were presented as mean ± SD. *p < 0.05, **p < 0.01, ***p < 0.001.

## Data Availability

Data sharing not applicable to this article as no datasets were generated or analysed during the current study.
