# Supplementary figures and images for: GATA1 Transcriptionally Upregulates LMCD1, Promoting Ferroptosis in Sepsis‐Associated Acute Kidney Injury Through the Hippo/YAP Pathway
Source: Kaohsiung J Med Sci. 2025 Jul 12;41(11):e70071. doi: 10.1002/kjm2.70071 (PMC12622420; doi:10.1002/kjm2.70071)

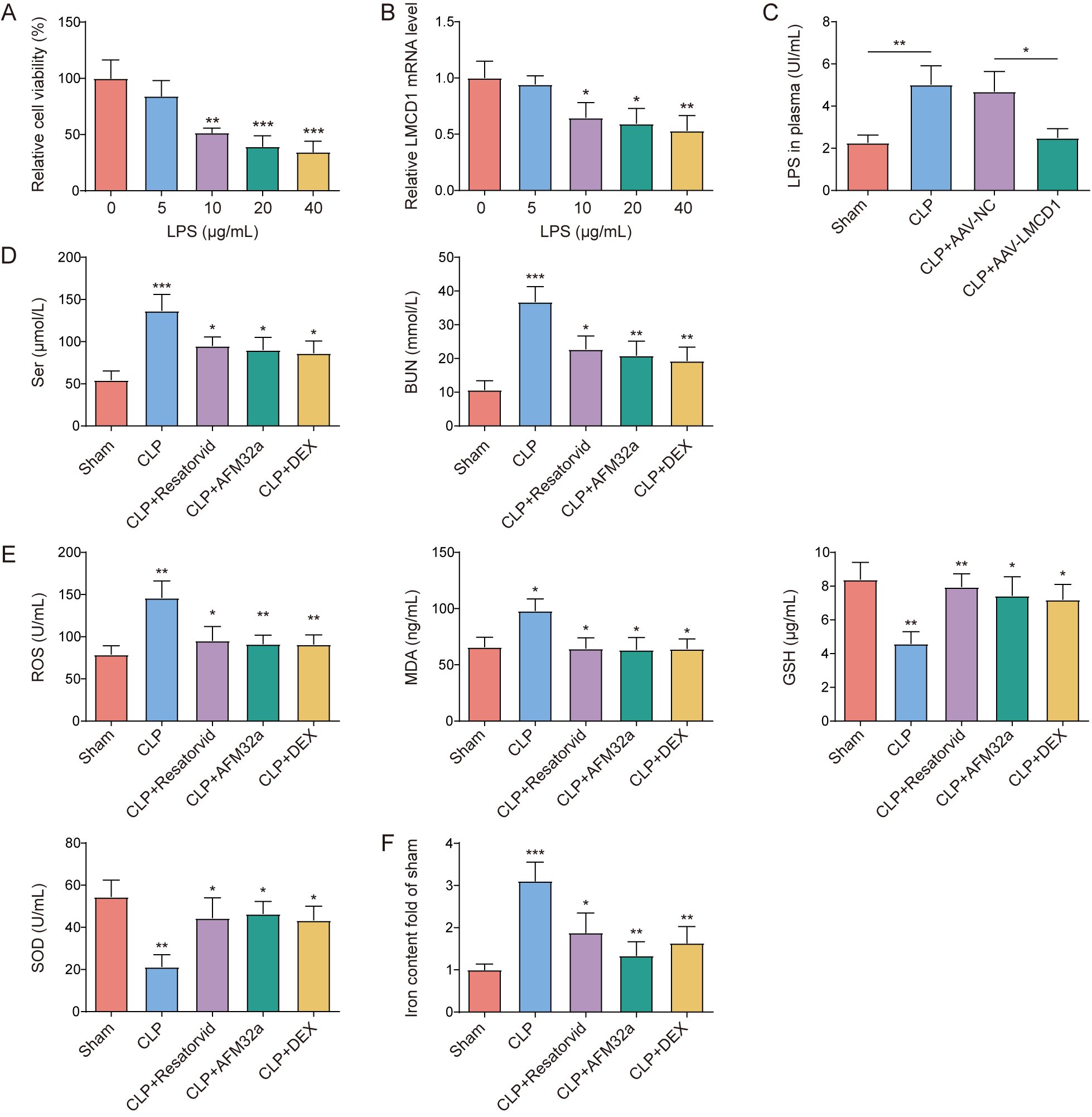

Supplement: Supplementary file 1 — Supporting Information Figure 1. HK‐2 cells were incubated with different concentrations of LPS (0, 5, 10, 20, and 40 μg/mL) for 22 h. (A) CCK‐8 assay was employed to detect HK‐2 cell viability. (B) LMCD1 mRNA level in cells was detected by qRT‐PCR. All data was obtained from at least three replicate experiments. SA‐AKI mice were established by CLP operation and then were injected with AAV‐LMCD1. (C) Plasma LPS level was measured by ELISA. SA‐AKI mice were established by CLP operation and then subjected to TLR4 inhibitor (Resatorvid, TAK‐242), PAD2 inhibitor (AFM32a), or DEX treatment. (D) Serum Scr and BUN levels were examined using ELISA. (E‐F) ROS, MDA, GSH, SOD, and Fe2+ levels in kidney tissues were analyzed by the kits. n = 6. The measurement data were presented as mean ± SD. *p < 0.05, **p < 0.01, ***p < 0.001. [file KJM2-41-e70071-s001.jpg]
